# Reduced level of arousal and increased mortality in adult acute medical admissions: a systematic review and meta-analysis

**DOI:** 10.1186/s12877-017-0661-7

**Published:** 2017-12-08

**Authors:** Amy Todd, Samantha Blackley, Jennifer K. Burton, David J. Stott, E. Wesley Ely, Zoë Tieges, Alasdair M. J. MacLullich, Susan D. Shenkin

**Affiliations:** 1 Medicine of the Elderly, NHS Lothian, Royal Infirmary, Edinburgh, Scotland; 2 Alzheimer Scotland Dementia Research Centre, Edinburgh, Scotland; 3 Centre for Cognitive Ageing and Cognitive Epidemiology, University of Edinburgh, Edinburgh, Scotland; 4 Institute of Cardiovascular and Medical Sciences University of Glasgow, Glasgow, Scotland; 5 Tennessee Valley Veteran’s Affairs Geriatric Research Education and Clinical Centre (GRECC), Nashville, TN USA; 6 Vanderbilt University Medical Centre, Nashville, TN USA; 7 Edinburgh Delirium Research Group, Geriatric Medicine, Edinburgh University, Edinburgh, Scotland

**Keywords:** Mortality, Altered mental status, Delirium, Systematic review, Glasgow Coma Scale

## Abstract

**Background:**

Reduced level of arousal is commonly observed in medical admissions and may predict in-hospital mortality. Delirium and reduced level of arousal are closely related. We systematically reviewed and conducted a meta-analysis of studies in adult acute medical patients of the relationship between reduced level of arousal on admission and in-hospital mortality.

**Methods:**

We conducted a systematic review (PROSPERO: CRD42016022048), searching MEDLINE and EMBASE. We included studies of adult patients admitted with acute medical illness with level of arousal assessed on admission and mortality rates reported. We performed meta-analysis using a random effects model.

**Results:**

From 23,941 studies we included 21 with 14 included in the meta-analysis. Mean age range was 33.4 - 83.8 years. Studies considered unselected general medical admissions (8 studies, *n*=13,039) or specific medical conditions (13 studies, *n*=38,882). Methods of evaluating level of arousal varied. The prevalence of reduced level of arousal was 3.1%-76.9% (median 13.5%). Mortality rates were 1.7%-58% (median 15.9%). Reduced level of arousal was associated with higher in-hospital mortality (pooled OR 5.71; 95% CI 4.21-7.74; low quality evidence: high risk of bias, clinical heterogeneity and possible publication bias).

**Conclusions:**

Reduced level of arousal on hospital admission may be a strong predictor of in-hospital mortality. Most evidence was of low quality. Reduced level of arousal is highly specific to delirium, better formal detection of hypoactive delirium and implementation of care pathways may improve outcomes. Future studies to assess the impact of interventions on in-hospital mortality should use validated assessments of both level of arousal and delirium.

**Electronic supplementary material:**

The online version of this article (10.1186/s12877-017-0661-7) contains supplementary material, which is available to authorized users.

## Background

### Rationale

Patients with reduced level of arousal on admission to hospital are common [1–5]. A range of scales are used to describe level of arousal; the Glasgow Coma Scale (GCS)[6], AVPU (Alert, responds to Verbal stimulus, responds to Painful stimulus and Unresponsive) [7], Observational Scale of Level of Arousal (OSLA) [8] and the Richmond Agitation-Sedation Scale (RASS) [9]. Reduced level of arousal is associated with mortality [10–12]. However, study populations and methods of assessment of level of arousal were heterogeneous.

Delirium is an acute, severe neuropsychiatric syndrome characterised by acute onset and fluctuating course, inattention and other changes in cognition, perceptual deficits, and altered level of arousal [13]. Delirium can be hyperactive - associated with increased activity and agitation - or hypoactive - associated with reduced level of arousal and lack of engagement, or mixed. Delirium is associated with poor outcomes [14–17] such as increased mortality: hazard ratio one year mortality for hyperactive delirium 1.3, hypoactive 1.6 and mixed 1.25 [18]. Many studies of delirium explicitly exclude people who are too drowsy to be tested [19], meaning studies of delirium and mortality are more difficult to interpret. Reduced level of arousal of acute onset, in the absence of trauma, is highly specific to delirium [4, 5, 8]. Hypoactive delirium is less likely to be recognised than cases with hyperactive features [2, 20, 21] and has poorer outcomes [18, 21, 22]. The majority of acute medical patients with reduced level of arousal are likely to have delirium, which may be undiagnosed, and the majority of these will be older patients. It is important to establish the association between reduced level of arousal and mortality.

### Objectives

We conducted a systematic review to establish if reduced level of arousal on admission to hospital with acute medical conditions is associated with increased mortality in adult patients.

## Methods

This review was reported in accordance with the Preferred Reporting of Items in Systematic Reviews and Meta-Analyses (PRISMA) guidance [23].

### Protocol and registration

The protocol was prospectively registered on Prospero: http://www.crd.york.ac.uk/PROSPERO/ (reference CRD42016022048).

### Eligibility criteria

The pre-determined inclusion criteria were (1) adults with acute medical illnesses requiring admission to hospital, (2) patients in emergency departments, acute medical units, acute receiving units, acute geriatric units, medical assessment units or equivalent, (3) patients in whom an assessment of level of arousal was made using either (i) a validated scale (e.g. GCS, AVPU, RASS or OSLA) or (ii) a subjective description (e.g. drowsy), (4) in-hospital mortality data comparing a drowsy group with a non-drowsy or less drowsy group.

The pre-determined exclusion criteria were (1) studies including children, (2) studies excluding patients aged over 65, (3) studies solely in intensive care units, (4) patients with a surgical condition given these patients may have undergone trauma, or early surgery and thus have exposure to anaesthetic agents, (5) studies solely including patients with direct central nervous system injuries: trauma, stroke, brain abscess, brain tumour, meningitis and encephalitis, (6) patients with poisoning, post drowning or post cardiac arrest. The protocol was altered to exclude patients with epilepsy and tropical diseases. Neither condition are typical of reduced level of arousal associated with general medical illness. If studies had a mixed population where less than half of the population had excluded conditions, these studies were included to reflect the case mix seen in general medical wards.

### Data sources

An inclusive search strategy was developed with an experienced librarian. The following data sources were searched in January 2016, and the search updated in June 2017: (1) Ovid MEDLINE (R) 1946 to present with daily update, (2) Ovid MEDLINE (R) In-process and other non-indexed citations, (3) Embase (1974 onwards) (Additional file 1: Appendix 1). We asked experts from the European Delirium Association and American Delirium Society to identify any additional references. The grey literature was not searched. We searched for articles in all languages and non-expertly translated potentially relevant abstracts where possible. We performed forward citation searches of included articles and checked reference lists of review articles.

### Data Collection

Two reviewers (AT, SB) independently reviewed all titles and abstracts for eligibility. They then independently evaluated full texts for inclusion, resolving any disagreement by discussion. Data extracted by each reviewer comprised: type of study, condition studied, age range with descriptive statistics setting, sample size, prevalence of drowsiness, arousal scale used to evaluate drowsiness and the definition used, descriptive terms used to describe level of arousal, in-hospital mortality in the two groups, any adjustments made to the analysis and the conclusion of the study. We primarily sought odds ratios (OR) for mortality. If these were not presented in the study, but the raw data were available, we calculated OR. Where there was ambiguity over results we contacted authors to clarify.

### Risk of Bias Assessment

Risk of bias for each study was assessed using a modified version of the Risk of Bias Assessment tool for Non-randomized Studies (RoBANS) [24] (Additional file 1: Appendix 2).

### Synthesis of Results

We performed quantitative analysis using Review Manager (RevMan) [25]. Dichotomous data were analysed using a random effects model to calculate a pooled OR with 95% confidence interval (CI). Statistical heterogeneity was quantified using I^2^ and supplemented by evaluation of the clinical heterogeneity and inspection of the forest plot. A sensitivity analysis was performed including only those studies which used the Glasgow Coma Scale to evaluate level of arousal.

## Results

### Study selection

**Fig. 1:**
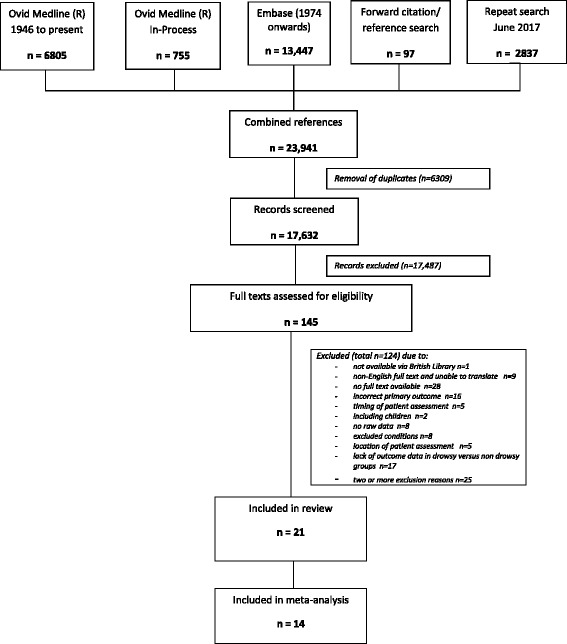
PRISMA flow chart of study selection

We identified 21,104 references, from which we sought 133 full texts in the initial search and 2837 references from which we sought 12 full texts in June 2017 (Fig. 1).

### Study characteristics

The review included 21 studies, eight of which comprised unselected medical admissions [4, 10–12, 26–29] and 13 which considered specific medical conditions [30–42]. All were cohort studies; 11 prospective and 10 retrospective. The 21 studies were published between 1990 and 2017 from Europe, Africa, North America, South America and Asia (Table 1). The sample size varied from 23 to 30,405 (median 469), with a total of 51,921 patients included in the review population. Mean ages ranged from 33.4 to 83.8 years. In studies with specific medical conditions the majority was respiratory (*n*=7), followed by endocrine (*n*=3), vasculitis (*n*=1), cardiology (*n*=1) and gastrointestinal (*n*=1). Several different scales were used to assess level of arousal: ten studies used GCS, two used AVPU, three used the Japan Coma Scale, one used the Kelly-Matthay scale and one used the Richmond Agitation and Sedation Scale (Additional file 1: Appendix 3). Three studies defined their own categories to describe level of arousal. Calle *et al* [34] described, but did not define, ‘altered level of consciousness’, however the paper made clear that these were patients with reduced level of arousal, rather than agitation. Eight studies did not present the proportion of patients with reduced level of arousal; in the remaining 13 studies the prevalence ranged from 3.1%-76.9% (median 13.5%). Mortality rates ranged widely, from 1.7%-58% (median 15.9%).

**Table 1:** Descriptive information from included studies of reduced level of arousal and mortality

Author and country	Year	Study type	Unselected medical/ disease specific	Mean age (SD)	Total N	Reduced Level of arousal n (%)	Total deaths n (%)	Arousal scale/ description
*Prospective studies using an arousal scale with unselected patients*								
Aslaner et al Turkey	2017	Prospective cohort	Unselected: ED admissions with altered mental status	77 (70-83) median with IQR	822	632 (76.9)	203 (24.7)	RASS: -4 and -5 versus -3,-2 and -1 versus 0,+1,+2,+3,+4
Rathour et al India	2015	Prospective cohort	Unselected: admissions with sepsis	50.5 (16.3)	200	46 (23)	116 (58)	GCS: ≤9 versus >9
Navinan et al Sri Lanka	2013	Prospective cohort	Unselected: medical unit admissions	50.7	167	Not given	10 (6.0)	GCS: mean
Francia et al Spain	2009	Prospective cohort	Unselected: admissions to medicine ward	73.6 (16.8)	500	Not given	65 (13)	GCS: mean
Burch et al S. Africa	2008	Prospective cohort	Unselected: medical admissions from ED	45.4 (17.0)	469	Not given	113 (24.1)	AVPU: A versus VPU
Duckitt et al England	2007	Prospective cohort	Unselected: medical admissions to an emergency unit	72.4 (range 17-106)	4286	Not given	355 (8.3)	AVPU: A versus VPU
*Prospective studies using an arousal scale with specific medical conditions*								
Nicolini et al Italy	2014	Prospective cohort	Respiratory: COPD	77.1	207	Not presented	33 (15.9)	Kelly Matthay Scale: From (1) alert to (6) comatose
Otieno et al Kenya	2010	Prospective cohort	Endocrine: Diabetic ketoacidosis	33.4 (15.2)	47	28 (59.6) (11 = GCS 9-12, 17 = GCS 3-6)	14 (29.8)	GCS: 13-15 versus 9-12 (drowsy) versus 3-8 (coma/ obtunded)
Dutta et al India	2008	Prospective cohort	Endocrine: Myxoedema coma	59.5 (14.8)	23	Not presented	12 (52.2)	GCS: mean
Delahaye et al France	2007	Prospective cohort	Cardiology: Infective endocarditis	59 (16.8)	559	Not presented	95 (17)	GCS: 9-15 versus 3-8
*Prospective studies not using an arousal scale for specific medical conditions*								
Calle et al Spain	2014	Prospective cohort	Respiratory: Community acquired pneumonia	85.4 (6.4)	456	61 (13.5)	110 (24.2)	‘altered level of consciousness’
*Retrospective studies using an arousal scale with unselected patients*								
Barfod et al Denmark	2012	Prospective cohort^b^	Unselected: admissions from ED	Not given	6279	197 ( 3.1)	107 (1.7)	GCS: ≤ 13 versus >13
Myint et al England	2011	Prospective cohort^a^	Unselected: medical nursing home admissions	83.8 (8.4)	316	Not given	78 (24.7)	GCS: cut-offs not presented
*Retrospective studies using an arousal scale for specific medical conditions*								
Sakamoto et al Japan	2017	Retrospective cohort	Respiratory: COPD	76 (8.9)	3064	393 (12.8)	209 (6.8)	JCS: alert, dull, somnolent, coma
Kaya et al Turkey	2016	Retrospective cohort	Gastrointestinal bleeding	62 (25)	600	21 (3.5)	38 (6.3)	GCS: 15 versus <15
Hasegawa et al Japan	2015	Retrospective cohort	Vasculitis: Churg Strauss	61.9 (15.6)	2195	96 (4.4)	97 (4.4)	JCS: alert versus non alert
Yamauchi et al Japan	2015	Retrospective cohort	Respiratory: Asthma/COPD/asthma-COPD mix	68.9 (14)	30405	2771 (9.1)	794 (2.6)	JCS: alert, dull, somnolent, coma.
Chih-Hsun et al Taiwan	2001	Retrospective cohort	Endocrine: Hyperglycaemic, hyperosmolar non-ketotic state	67.8 (11.7)	119	89 (74.8) (54 GCS 9-14, 38 GCS 3-8)	29 (24.4)	GCS: mean
*Retrospective studies not using an arousal scale for specific medical conditions*								
Conte et al USA	1999	Retrospective cohort	Respiratory: Community acquired pneumonia	Not given, all over 65	1000	80 (8)	87 (8.7)	Eye opening: spontaneous versus not. Verbal: orientated versus not. Motor: to voice versus not
Onadeko et al Kuwait	2005	Retrospective cohort	Respiratory: COPD	63.7 (12.6)	74	18 (24.3)	14 (18.9)	Descriptive terms: ‘drowsy’ versus ‘alert’
Zweig et al USA	1990	Retrospective cohort	Respiratory: Pneumonia	80 (no SD)	133	65 (48.9) non-alert	21 (15.8)	Categories: alert, confused, arousable, comatose

^a^paper stated both prospective and retrospective- prospective data collection but reviewed the information retrospectively

^b^paper stated prospective, but we considered it to be retrospective as it extracted data from a previously populated database

Level of arousal: Level of Arousal

*JCS* Japan Coma Scale

### Risk of bias

**Fig. 2:**
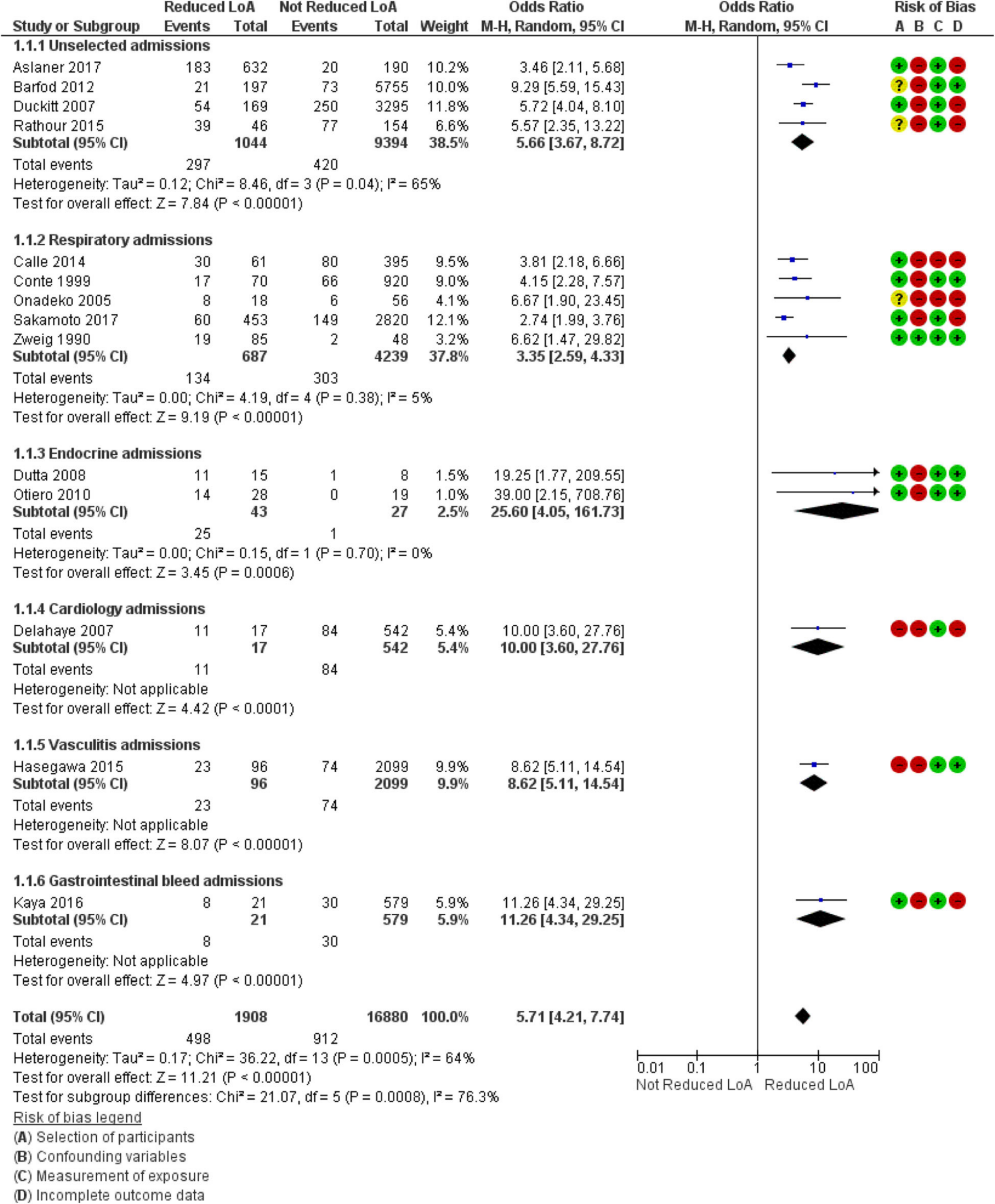
Forest plot of association between level of arousal and mortality

Risk of bias was generally high for the consideration of confounding variables (Fig. 2), with a lack of sufficient information in individual studies regarding features such as the presence of dementia, use of sedative drugs, psychoactive drugs or alcohol, or inclusion of these features in multivariate analyses. The risk of confounding bias was only deemed low in one study [40], which considered both dementia and psychoactive medication use. Selection of patients and incomplete data outcome were variable (Additional file 1: Appendix 4). Most studies used a known scale to measure level of arousal, therefore the risk of bias from measurement of exposure was low, although training in using the scales was not reported. None of the studies had published a protocol, therefore the risk of selective outcome reporting was unknown.

### Quantitative results

A wide range of scales were used to assess level of arousal. Different cut-offs were used to define the categories of drowsy and non-drowsy/less drowsy making direct comparison impossible across included studies. Multivariate analysis was performed on 16 of the studies; however, the potential confounding factors included in the analysis varied greatly (Additional file 1: Appendix 5). Raw data was available for 12 studies and two authors [12, 33] provided raw data to allow inclusion in meta-analysis.

Reduced level of arousal was associated with increased inpatient mortality (pooled OR 5.71 95% CI 4.21-7.74; 21,198 patients, low quality evidence: risk of bias, clinical heterogeneity, risk of publication bias). There is significant heterogeneity with an I^2^ of 64%. This can be explained by the variation in medical conditions studied and range of scales used. Two further studies[29, 36] reported the association between reduced level of arousal and mortality as: OR 5.10 95% CI 3.10-8.39, OR 5.65 95% CI 3.35-9.53, event rates were not available so these data could not be pooled.

Sensitivity analysis including only those studies using GCS confirmed the direction of the observed effect with a pooled OR of 9.16 (95% CI 6.37-13.18; 7,381 patients, low quality evidence due to risk of bias and clinical heterogeneity).

Data from a further five studies could not be pooled as there was insufficient data available to calculate a univariate OR [11, 26, 28, 30, 37]. In patients with COPD survivors had a lower mean score (2) using the Kelly Matthay Scale [30] (see Additional file 1: Appendix 3) than those who died. Nursing home patients with medical admissions[28] reported “GCS at the time of admission was significantly associated with in-patient death”: OR 0.877 (99% CI 0.792-0.970), that is, higher GCS was associated with reduced mortality. On multivariate analysis the hazard ratio for survival for ‘low GCS’ was 0.924 (99% CI 0.880-0.970). Mean GCS levels were 11.8 (+/-3.2) in survivors with hyperglycaemic, hyperosmolar non-ketotic states [37] versus 7.7 (+/-4.3) for those who died in-hospital (95% CI of the difference -5.8 to -2.3). On multiple logistic regression low GCS on admission was the only factor contributing to death (OR 14.012 *p*<0.001 (no CI given)). A cohort of unselected patients[11]found mean GCS of 14 (+/-2) in survivors and 13 (+/-3) in non-survivors. On multivariate analysis GCS was reportedly an independent predictor of mortality: OR 0.883 (95% CI 0.790-0.988) which was the OR of higher GCS and mortality. A preliminary study looking at early warning scores (EWS) [26] showed mean GCS was 13.1 in those who died versus 14.8 in survivors, *p*=0.2330. No multivariate analyses were performed. This was the only study which did not find a statistically significant association between reduced level of arousal and in-hospital mortality. It was however small (*n*=167) with only ten deaths and the risk of bias was either high or unclear in all categories.

### Risk of bias across studies

The funnel plot (Additional file 1: Appendix 6) suggests that there may be publication bias against negative small studies.

## Discussion

### Summary of findings

This systematic review and meta-analysis demonstrates that acute medical patients with reduced level of arousal on admission to hospital have a substantially higher risk of mortality compared with those with normal or heightened level of arousal. The meta-analysis, performed using 14 of the 21 studies, found reduced level of arousal was associated with a 5.7-fold increased risk of in-hospital mortality. We felt it was important to perform a meta-analysis on these studies to confirm the underlying effect size. Sensitivity analysis including only those studies using GCS- the most widely used clinical arousal test, which has been in use without change for several decades- confirmed the direction of the observed effect with a pooled OR of 9.16. This was performed to reduce the degree of heterogeneity but note significant clinical heterogeneity remains. Studies not included in the meta-analysis showed results in the same direction, but some upper confidence intervals were close to one, suggesting some overlap between the group. This occurred in three studies. These studies were generally small and used different cut-offs to determine low and high GCS. Meta-regression was not performed due to heterogeneity of studies.

These findings have important caveats in that the included studies were heterogeneous in the populations studied and methods used to measure level of arousal. Although overall we considered the available evidence to be of low quality the consistency between studies in demonstrating a positive association between reduced level of arousal and mortality and the narrow CI for the pooled data is notable.

Delirium is also associated with increased mortality [15–18] and the majority of patients with acute-onset reduced level of arousal meet criteria for delirium [4, 5, 8, 13]. Additionally, some delirium studies exclude patients with severely reduced level of arousal [19]; this restricted spectrum may have led to underestimation of the relationship between delirium diagnosis and mortality.

The 5.7-fold mortality rate can be compared with other illness severity indicators: for example raised lactate (>4 mmol/L) on admission to hospital has an OR for in hospital mortality of four (95% CI 1.7-14.1) [43–45] and hypotension <100mmHg has an OR of 2.0 (95% CI 1.3-2.8) [46] and <90mmHg OR 3.88 (95% CI 2.62-5.75) [47].

### Strengths of the review

This was a large and comprehensive systematic review evaluating over 23,000 references using an inclusive search strategy. All references, abstracts and full texts were assessed by two independent reviewers. We translated articles as able, followed up conference abstracts and performed forward citation searches. We contacted authors for data and clarification. In light of predicted significant heterogeneity, a random effects model was used in the meta-analysis.

### Limitations of the review

It is possible that relevant studies could have been missed. We did not include non-published studies or the grey literature. We were able to translate five non-English studies but there remained nine we could not translate. No full text was available for 28 abstracts; mainly conference abstracts. We searched for future publication of full text for these articles but none were identified. Not all studies presented the data required to calculate OR but available data was increased following correspondence with study authors. Another three study authors attempted to retrieve their raw data but were unable. The available evidence from the studies included in this review was considered of low quality overall, due to the risk of bias, clinical heterogeneity and the risk of publication bias. However, similar results were found if studies that were retrospective and/or used no validated arousal scale, were removed.

### Interpretation and implications for clinical practice and further research

No previous systematic review has explored the relationship between reduced level of arousal and mortality. We were unable to explore the reasons underpinning this association. It is possible that patients with reduced level of arousal had more severe illness, however, multivariate analyses suggest reduced level of arousal is still associated with increased mortality after correcting for vital signs, and thus this is unlikely to be the sole explanation. It is plausible that reduced level of arousal contributes causally to poor outcomes, through increased risk of aspiration pneumonia, increased practical challenges of providing medical care, and impairing the ability to undergo rehabilitation.

The poor prognosis of delirium is increasingly recognised [14–17]. The majority of studies did not present sufficient information to allow us to comment on the presence of delirium, but it is established that acute-onset reduced level of arousal, in non-comatose patients, is a highly specific indicator of delirium [4, 5, 8, 13]. Only two [4, 34] of the included studies looked for delirium amongst their patients. Many studies of delirium specifically exclude patients with reduced level of arousal [19]. Given the 5.7-fold increased risk of in-hospital mortality in this group clinicians need to be vigilant regarding these patients, consider discussion around prognosis with patients and families, and actively seek evidence to diagnose delirium and manage it appropriately.

Future research should examine the outcomes of both reduced level of arousal and delirium, considering likely aetiologies and causes of death. This would require prospective cohort studies evaluating sufficient numbers of patients, including those with primary neurological disease and/or surgical conditions for predetermined sub-group analyses. Validated level of arousal scales should be used rather than descriptive terms. Comprehensive characterisation of patient demographics, co-morbidities including dementia, drugs (particularly use of psychoactive or sedative drugs) and alcohol use should be reported. Delirium studies should include patients who are too drowsy to undergo cognitive testing or interview. This could be achieved by using specific level of arousal assessment instruments, or by using delirium scales with embedded level of arousal measurement such as the 4 “A”s Test (4AT) [48].

## Conclusions

In this systematic review and meta-analysis, reduced level of arousal on admission to hospital with general medical illnesses is associated with a 5.7-fold increased risk of in-hospital mortality. Patients with reduced level of arousal should therefore be identified as having a high risk of in-hospital death, and their care should take this into account. As acutely reduced level of arousal is a strong indicator of delirium, patients with reduced level of arousal should be assessed for delirium, and follow a delirium management pathway if diagnosed.

## Additional file

Additional file 1:
**Appendix 1**. Search terms used in Medline search. **Appendix 2**. Modified RoBANS risk of bias assessment. Description of risk of bias assessment undertaken. **Appendix 3**. Description of level of arousal scales. **Appendix 4**. Risk of bias assessment for included studies. **Appendix 5**. Results of association between level of arousal and mortality. Table of data from included studies. **Appendix 6**. Funnel plot of thirteen published studies which contribute to meta-analysis. (DOCX 39 kb)

## Glossary

AVPU: Alert, responds to Verbal stimulus, responds to Painful stimulus and Unresponsive
CI: Confidence Interval
COPD: Chronic Obstructive Pulmonary Disease
ED: Emergency Department
EWS: Early Warning Scores
GCS: Glasgow Coma Scale
IQR: Inter Quartile Range
JCS: Japan Coma Scale
OR: Odds Ratio
OSLA: Observational Scale of Level of Arousal
PRISMA: Preferred Reporting of Items in Systematic Reviews and Meta-Analyses
RASS: Richmond Agitation-Sedation Scale
RoBANS: Risk of Bias Assessment tool for Non-randomized Studies
SD: Standard Deviation
