# Supplementary material for: Reduced level of arousal and increased mortality in adult acute medical admissions: a systematic review and meta-analysis
Source: BMC Geriatr. 2017 Dec 8;17:283. doi: 10.1186/s12877-017-0661-7 (PMC5721682; doi:10.1186/s12877-017-0661-7)

**Appendix 1: Search terms**

1. consciousness disorders/

2. drows*.ti,ab.

3. glasgow coma scale/

4. GCS.ti,ab.

5. glasgow coma scale.ti,ab.

6. AVPU.ti,ab.

7. conscious*.ti,ab.

8. level adj2 arousal.ti,ab.

9. RASS.ti,ab.

10. Richmond adj3 Scale.ti,ab.

11. OSLA.ti,ab.

12. 1 or 2 or 3 or 4 or 5 or 6 or 7 or 8 or 9 or 10 or 11

13. mortality/ or hospital mortality/

14. mortalit*ti,ab.

15. prognosis.ti,ab.

16. 13 or 14 or 15

17. 12 and 16

**Appendix 2: Modified RoBANS risk of bias assessment**

This involves an assessment of whether the risk of bias in an observational study is ‘high’, ‘low’ or ‘unknown’ over six domains:

(A) Selection of participants. We assessed how comprehensive the attempt was to include all relevant patients in the study. Low risk of bias studies had a thorough methodology to ensure the maximum number of eligible patients were included.

(B) Confounding variables. We recognised that multiple confounding factors could contribute to a patient being drowsy on admission to hospital, particularly features such as use of psychoactive or sedative drugs, or alcohol use. If such features were documented and included in a multivariate analysis, the study was deemed to be low risk.

(C) Measurement of exposure. For each study we assessed the robustness of the arousal assessment. Use of a validated scale (e.g. GCS) was considered low risk whereas descriptive terms (e.g. ‘impaired consciousness’) were high risk.

(D) Incomplete data outcome. We assessed the risk of bias based on how missing data were handled. If over 10% of patients had to be excluded due to missing data, or if there was no comment on handling of missing data, these studies were deemed high risk.

(E) Blinding of outcome. Given our outcome was death this was not relevant for our studies.

(F) Selective outcome reporting. If a protocol was available we read this to determine whether the study followed the pre-specified plan. If there was no protocol it was deemed unclear.

**Appendix 3: Description of level of arousal scales**

Glasgow Coma Scale

Eye response: Spontaneous = 4

Verbal = 3

Pain = 2

None = 1

Verbal response: Orientated = 5

Confused = 4

Inappropriate words = 3

Incomprehensible sounds = 2

None = 1

Motor response: Obeys commands = 6

Localises painful stimulus = 5

Withdraws from pain = 4

Flexion to pain = 3

Extension to pain = 2

None = 3 Total score out of 15, minimum score 3

AVPU

A = alert

V = responds to vocal stimuli

P = responds to pain

U = unresponsive

Japan Coma Scale

A state of not awakening even when stimulated (deep coma, coma, semi-coma):

- 300 No response to pain

- 200 Moves hands or feet slightly to pain

- 100 Withdrawal or localisation to painful stimulus

A state of awakening to stimulus (stupor, lethargy, hypersomnia, somnolence, drowsiness):

- 30 Opening eyes to a repeated call and painful stimulus

-20 Opening eyes to shaking body or shouting

- 10 Opening eyes to ordinary voice

A state of awakeness without stimulation (delirium, confusion, senselessness)

- 3 Cannot state name or date of birth

- 2 Disorientation

- 1 Consciousness not completely clear

Kelly Matthay Score

1 Alert, following complex three stage commands

2 Alert, follows simple commands

3 Lethargic but arousable and follows simple commands

4 Stuporous, only intermittently follows simple commands

5 Comatose, brain stem intact

6 Comatose with brain stem dysfunction

Richmond Agitation and Sedation Scale

+4 Combative

+3 Very agitated

+2 Agitated

+1 Restless

0 Alert and calm

-1 Drowsy

-2 Light lethargy

-3 Moderate lethargy

-4 Deep lethargy

-5 Unarousable

**Appendix 4: Risk of bias assessment for included studies**


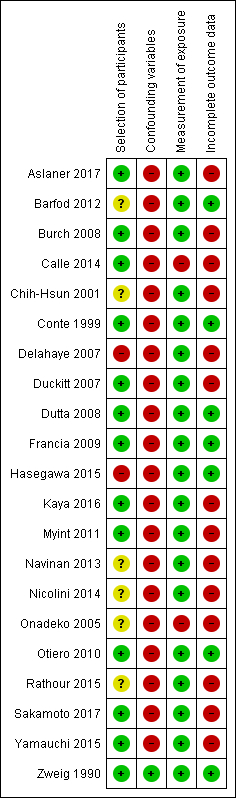


As our outcome was death we did not consider that blinding of this outcome was an important source of potential bias, therefore this was not included.

**Appendix 5: Results of association between level of arousal and mortality**

| **Author** | **Definition of reduced Level of arousal** | **Univariate odds ratio (OR) for in-hospital mortality, or equivalent** | **Multivariate OR** | **Covariates included in multivariate analysis** | **Other results / comments** |
| --- | --- | --- | --- | --- | --- |
| ***Prospective studies using an arousal scale with unselected patients*** | | | | | |
| Aslaner *et al* | RASS: -4 and -5 versus -3,-2 and -1 versus 0,+1,+2,+3,+4 | OR 3.46 (95% CI 2.11-5.68) p<0.0001 | No multivariate analysis performed. | NA |  |
| Rathour *et al* | GCS≤9 | OR 5.57 (95% CI 2.35-13.22), p=0.0001 | 6.02 (95% CI 2.24-16.19), p=0.001 | age, COPD, chronic liver disease, cancer, presence of catheter, type of infection, BP, oxygen saturations, urine output, APTT and creatinine | Heterogeneous group, 48.5% history of alcoholism, 35% of drug abuse but these factors were not included on multivariate analysis. |
| Navinan *et al* | Mean GCS | Insufficient data to calculate ORs. Mean GCS: 13.1 in those who died versus 14.8 in survivors, p=0.2330. | No multivariate analysis performed. | NA | Small study with only ten deaths and breakdown of medical diagnoses not presented. |
| Francia *et al* | Mean GCS | Insufficient data to calculate ORs. Mean GCS in survivors: 14 (SD 2) Mean GCS in non-survivors: 13 (SD 3) | 0.883 (95% CI 0.790-0.988), p=0.030 | age, mean arterial pressure, heart rate, respiratory rate and oxygen saturations |  |
| Burch *et al* | Verbal/Pain/Unresponsive (versus Alert) | Insufficient data to calculate ORs. | 5.1 (95% CI 3.1-8.3) no p value | systolic BP, pulse, respiratory rate and temperature |  |
| Duckitt *et al* | Verbal/Pain/Unresponsive (versus Alert) | Insufficient data to calculate ORs. | 3.50 (95% CI 2.30-5.32), p<0.001. | respiratory rate, heart rate, BP, temperature and oxygen saturations |  |
| ***Prospective studies using an arousal scale with specific medical conditions*** | | | | | |
| Nicolini *et al* | Kelly Matthay Scale:  From (1) alert to (6) comatose | Insufficient data to calculate ORs. | mean (SD) score was 2 (1) in survivors and 3 (1) in those who died, p=0.01 | sex, age, NIV settings, comorbidities and gas exchange | Multiple stepwise regression. The number of drugs taken per day was recorded but not included in multivariate analysis. |
| Otieno *et al* | GCS: 13-15 versus 9-12 (drowsy) versus 3-8 (coma/obtunded) | OR 39, (95% CI 2.15-708.80), p=0.0133 | No multivariate analysis performed. | NA | Reduced Level of arousal was significantly associated with systolic hypotension and severe metabolic acidosis. All patients with reduced Level of arousal died. |
| Dutta *et al* | Mean GCS | OR 19.25 (95% CI 1.77-209.55) p=0.0152 |  |  | Multiple linear regression model (including method IV T4, GCS and SOFA score), lower GCS was associated with increased mortality (r=0.45, p=0.03). All patients who died were on sedatives but this wasn’t included in the multiple regression model. |
| Delahaye *et al* | GCS≤8 | GCS 9-15: 15.5% died  GCS 3-8: 64.7% died, p<0.0001  Insufficient data to calculate ORs. | 4.09 (95% CI 1.17-14.36), no p value | age, gender, comorbidities, cardiac history, at-risk procedures, echocardiography findings, microorganism, severity signs, vascular and immunological phenomena and signs of infection | Some ‘classical’ risk factors e.g. age were not significant on multivariate regression in this study. Data combined from two databases 8 years apart – it would be expected that over that timescale the medical management improved. |
| ***Prospective studies not using an arousal scale for specific medical conditions*** | | | | | |
| Calle *et al* | ‘altered level of consciousness’ | OR 3.81 (95% CI 2.18-6.66), p<0.0001 | 3.197 (95% CI 1.66-6.15) p=0.001 | age, sex, Barthel index, Lawton index, haematocrit, pleural effusion and multilobar involvement |  |
| ***Retrospective studies using an arousal scale with unselected patients*** | | | | | |
| Barfod *et al* | GCS≤13 | OR 9.29 (95% CI 5.59-15.44), p<0.0001 | GCS 9-13: OR 3.72 (95% CI 1.97-7.03), p<0.0001  GCS <8: OR 10.97 (95% CI 4.90-24.56), p<0.0001 | age, oxygen saturations, respiratory rate and systolic BP |  |
| Myint *et al* | ‘Low GCS’ | OR 0.877 (99% CI 0.792-0.970), p=0.01 | Hazard ratio 0.924 (99% CI 0.880-0.970), p<0.0001 | age, gender, systolic BP, temperature and respiratory rate | Multiple Cox-regression model |
| ***Retrospective studies using an arousal scale for specific medical conditions*** | | | | | |
| Sakamoto *et al* | JCS: alert, dull, somnolent, coma | OR 2.74 (95% CI 1.99-3.76) p<0.0001 | Dull OR 1.59 (95% CI 1.04-2.44) p=0.032  Somnolent OR 1.71 (95% CI 0.8-3.65) p=0.168  Comatose OR 4.32 (95% CI 2.29-8.15) p<0.001 | Age, gender, BMI, dyspnoea, mechanical ventilation, pneumonia, asthma, heart failure | Multivariate logistic regression. |
| Kaya *et al* | GCS: 15 versus <15 | OR 11.26 (95% CI 4.34-29.25) p<0.0001 | No multivariate analysis performed. | NA |  |
| Hasegawa *et al* | JCS alert versus not alert | OR 8.62 (95% CI 5.11-14.54) p<0.0001 | 3.94 (95% CI 2.2-7.07), p<0.001 | age, sex, type of admission, co-morbid disease and sepsis | Multivariable logistic regression. No mention of dementia as a co-morbidity. None of the patients with ENT disease (n=130) died and these patients were excluded from multivariate analysis. |
| Yamauchi *et al* | JCS alert, dull, somnolent, coma | Insufficient data to calculate ORs. | ‘Dull’ OR 1.3 (95%CI 0.90-1.88), p=0.158; ‘Somnolent’ OR 3.1 (95%CI 1.91-6.10), p<0.001  ‘Coma’ OR 5.65 (95%CI 3.35-9.51), P=0.01. | age, gender, BMI, dyspnoea grade, Barthel index, daily steroid doses and co-morbidities including anxiety and depression | Multivariate logistic regression |
| Chih-Hsun *et al* | Mean GCS | Mean (SD) GCS in those who died 7.7 (4.3) versus 11.8 (3.2) in survivors (95% CI of the difference -5.8 to -2.3), p<0.001. Insufficient data to calculate ORs. | 14.012 p<0.001. (no CI given). | age, gender, smoking and biochemistry | Multiple logistic regression. Categories of GCS given in methods describe GCS 15 as ‘mild’, but all results are mean GCS so categories likely irrelevant. |
| ***Retrospective studies not using an arousal scale for specific medical conditions*** | | | | | |
| Conte *et al* | Eye opening: spontaneous versus not. Verbal: orientated versus not. Motor: to voice versus not. | Eye opening OR 4.1 (95% CI 2.8-7.57) p <0.0001  Verbal response OR 3.61 (95% CI 2.28-5.73), p<0.0001  Motor response OR 4.21 (95% CI 2.36-7.52), p<0.0001 | Impaired motor response OR 2.3 (95% CI 1.4-3.7) p=0.0009. | age, co-morbid disease, abnormal vital signs and high creatinine | Co-morbidities of dementia or presence of delirium not sought.  Patients who died on the day of admission were excluded. |
| Onadeko *et al* | Descriptive terms: alert versus drowsy | OR 6.67 (95% CI 1.9-23.4), p=0.0031 | No multivariate analysis performed. | NA | The definition of drowsy is unclear. The OR presented in the paper are the odds of survival. We have calculated ORs for in-hospital mortality. |
| Zweig *et al* | Categories: alert, confused, arousable, comatose | OR 6.62 (95% CI 1.47-29.81), p=0.0138 | 11.3 (95% CI 3.2-40.3). No p value given. | high respiratory rate, low temperature, leucocytosis, cyanosis, coronary artery disease, dementia and urinary incontinence | Stepwise logistic regression. Those who died were significantly more likely to have dementia (no OR presented, p<0.01). Use of psychoactive drugs in those who survived and those who died was considered and found not to be associated with mortality (but no statistics presented). |

**Appendix 6 Funnel plot of thirteen published studies which contribute to meta-analysis**


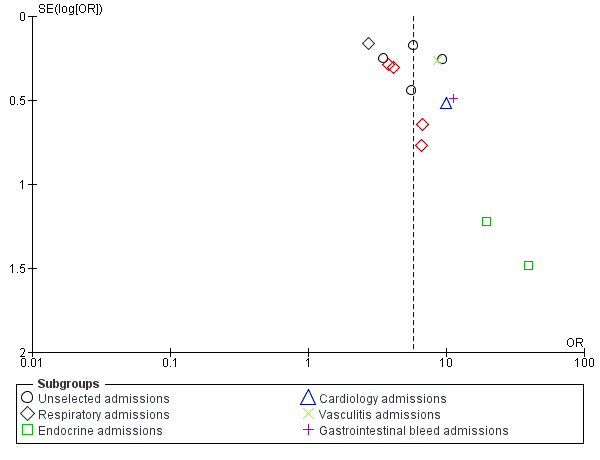

Supplement: Additional file 1: — Appendix 1. Search terms used in Medline search. Appendix 2. Modified RoBANS risk of bias assessment. Description of risk of bias assessment undertaken. Appendix 3. Description of level of arousal scales. Appendix 4. Risk of bias assessment for included studies. Appendix 5. Results of association between level of arousal and mortality. Table of data from included studies. Appendix 6. Funnel plot of thirteen published studies which contribute to meta-analysis. (DOCX 39 kb) [file 12877_2017_661_MOESM1_ESM.docx]
